# *‘People say that we are already dead much as we can still walk’:* a qualitative investigation of community and couples’ understanding of HIV serodiscordance in rural Uganda

**DOI:** 10.1186/s12879-016-1998-9

**Published:** 2016-11-10

**Authors:** Jiho Kim, Mastula Nanfuka, David Moore, Murisho Shafic, Maureen Nyonyitono, Josephine Birungi, Florence Galenda, Rachel King

**Affiliations:** 1 BC Centre for Excellence in HIV/AIDS, Vancouver, BC Canada; 2 The AIDS Support Organization, Jinja, Uganda; 3 Faculty of Medicine, University of British Columbia, Vancouver, BC Canada; 4 Global Health Sciences, University of California San Francisco, San Francisco, California, USA

**Keywords:** HIV, Sexual health, Serodiscordance, Uganda, Africa

## Abstract

**Background:**

Stable, co-habiting HIV serodiscordant couples are a key population in terms of heterosexual transmission in sub-Saharan Africa. Despite the wide availability of antiretroviral treatment and HIV educational programs, heterosexual transmission continues to drive the HIV epidemic in Africa. To investigate some of the factors involved in transmission or maintenance of serodiscordant status, we designed a study to examine participants’ understanding of HIV serodiscordance and the implications this posed for their HIV prevention practices.

**Methods:**

In-depth interviews were conducted with 28 serodiscordant couples enrolled in a treatment-as-prevention study in Jinja, Uganda. Participants were asked questions regarding sexual behaviour, beliefs in treatment and prevention, participants’ and communities’ understanding and context around HIV serodiscordance. Qualitative framework analysis capturing several main themes was carried out by a team of four members, and was cross-checked for consistency.

**Results:**

It was found that most couples had difficulty explaining the phenomenon of serodiscordance and tended to be confused regarding prevention. Many individuals still held beliefs in pseudoscientific explanations for HIV susceptibility such as blood type and blood “strength”. The participants’ trust of treatment and medical services were well established. However, the communities’ views of both serodiscordance and treatment were more pessimistic and wrought with mistrust. Stigmatization of serodiscordance and HIV-positive status were reported frequently.

**Conclusions:**

The results indicate that despite years of treatment and prevention methods being available, stigmatization and mistrust persist in the communities of HIV-affected individuals and may directly contribute to new cases and seroconversion. We suggest that to optimize the effects of HIV treatment and prevention, clear education and support of such methods are sorely needed in sub-Saharan African communities.

## Background

Expanded access to antiretroviral treatment (ART) has significantly reduced HIV-associated mortality and has contributed to reduced HIV incidence, even in the most highly affected region, sub-Saharan Africa. An estimated 1.4 million new infections occurred in the region in 2014, which account for approximately 70 % of new infections worldwide [1]. Most new infections in this region are due to heterosexual transmission, which distinguishes the epidemic in this area from most other regions of the globe where transmission is driven predominantly by transmission between men who have sex with men (MSM) or people who use injection drugs. Transmission within HIV serodiscordant couples in marriage or cohabitation is thought to account for the majority of new infections [2–4]. This major difference necessitates the need for clear understanding of HIV-serodiscordant relationships and the couples’ understanding of HIV, serodiscordance, prevention, and treatment.

Much of the research and program effort in HIV prevention for serodiscordant couples in Africa thus far has centred on the correct use of condoms [5–7], microbicides [8, 9], the possibility of treatment as prevention (TasP) [10–12], and voluntary counselling and testing (VCT) [13, 14]. Many of these studies were able to establish the effectiveness of such population-based methods in reducing HIV incidence [15, 16], but it still remains clear that all of these interventions are dependent on behaviour and decision-making on part of the affected individuals. Thus, it is important to understand how individuals involved in a serodiscordant relationship perceive their status and the interventions taking place in their community.

With the publication of the HPTN 052 trial which demonstrated a 96 % reduction in HIV transmission within serodiscordant couples [17] associated with early use of ART, HIV treatment in conjunction with proper education and awareness [18, 19] are now recommended as best practice for preventing transmission within serodiscorant relationships. Effectiveness of such interventions depend heavily on the couples’ attitudes toward such interventions and treatment; this has been more difficult to measure but much misinformation and distrust persists in both the serodiscordant populations and their surrounding communities [20–22]. Previous qualitative studies have found beliefs in “stronger blood” [23] and “spirits and supernatural forces” [24] behind the infection, all of which may contribute to some of the behavioural challenges cited above [25] and may undermine prevention efforts.

We carried out a qualitative study to explore the perceptions of members of HIV serodiscordant couples in terms of their understanding of serodiscordance or eventual seroconversion. Furthermore, we examined how this understanding affected and their sexual behavior and adherence to ART (for positive participants) in the context of an observational study of TasP in rural east-central Uganda.

## Methods

All of the HIV positive participants were clients of The AIDS Support Organization (TASO) in Jinja, Uganda. TASO is the oldest and largest non-governmental HIV care and treatment organization in Uganda. TASO provides treatment and support to over 100,000 HIV-affected clients through 11 service centres across the country. The ART program at TASO-Jinja began in 2004. Jinja is a moderate-sized town (population 90 000) in the eastern region of Uganda, approximately 80 km east of the capital, Kampala. Since serodiscordance was recognized as an important contributor to the HIV epidemic in Uganda, TASO has provided opportunities for discordant couples to participate in support groups at all TASO centres as part of their routine programming. Thus, all study participants became TASO clients. As well, the concept of TasP has been widely reported in the Ugandan media since the publication of the results of the HPTN 052 trial in 2010, but it is not known how widely this concept has diffused down to the local level to HIV care and treatment programs. The 2013 WHO guidelines have been largely adopted by Uganda in terms of recommending ART for HIV positive individuals whose regular sexual partners are HIV negative [26]. However these guidelines had not yet been formulated at the time of this study.

Study subjects were part of an observational study of TasP among serodiscordant couples known as the Highly Active Antiretroviral therapy as Prevention (HAARP) study. The HAARP study compared HIV incidence between HIV negative members of serodiscordant partners who were or were not receiving ART during the study and was conducted from June 2009 to December 2011. The study did not find a benefit from ART in terms of preventing HIV seroconversion in the negative participant [27]. As a result of these findings, we enrolled a selection of previous study participants in a qualitative sub-study for a series of in-depth interviews beginning in June 2013 and continuing until August 2014.

A total of 28 couples, or 57 individuals were recruited for the study. At recruitment for this sub-study, one “couple” was involved in a polygamous relationship (one husband and two wives). All couples were initially serodiscordant upon recruitment into the HAARP study. However, over the course of the HAARP study 14 of the HIV negative participants seroconverted. We specifically selected these individuals and their partners for this study. We then recruited 14 age (within +/- 5 years) and gender-matched control HIV positive participants whose partners did not seroconvert in the study.

Beginning in June 2013, trained interviewers carried out five individual in-depth interviews over 12 months, with each member of the couple interviewed separately. Interviews were conducted at the TASO clinic site at Jinja, Uganda. The interviewers were a diverse group of TASO caregivers, comprised of 1 physician, 2 nurses, and one counsellor. Participants were compensated with 20 000 Ugandan shillings (approximately $8 USD) at each interview for transportation costs. All interviews were gender-matched between the participant and the interviewer. Interviewers asked open-ended questions regarding the couples’ perceptions of serodiscordance, the participants’ views of the community’s perception of serodiscordance, the couples’ sexual behaviour, desire to have children, opinions regarding HIV prevention strategies and the role in which HIV treatment was incorporated into these strategies. For those couples where seroconversion occurred, further discussion included their perceptions of why this occurred. The interviews were carried out in the preferred local language (Luganda or Lusoga) of the participants, recorded, and then transcribed and translated into English. We report here on the first of the five interviews.

Three trained analysts coded translated transcripts using NVivo software for data management. We used for thematic coding and framework analysis as the primary analytic strategy. Framework analysis uses four analytic stages: familiarization (reading multiple times), identification of themes (developing the codebook), indexing and charting that involves arranging summaries of the data into a database according to theme, sub-theme, category and interpretation [28]. Thus, after reading three transcripts, the analysis team members collaboratively developed a codebook of themes based on the interview topics as well as those emerging from the data. Three more transcripts were then reviewed to include additional topic areas and themes. This process was repeated until the codebook reached a stage where no new themes or topic areas emerged. All transcripts were then coded using the final version of the codebook before themes were summarized across respondents. Analysis focused on identifying the dominant themes and the range of explanations for sexual behavior, motivations for preventive behaviour, treatment and comparisons across participants. Interactive discussions were held with the analysis team to validate data interpretations and resolve any interpretation discrepancies.

The analysis team regularly compared coding to ensure inter-rater consistency. NVivo Software Version 10 (QSR International, Victoria, Australia) was used for all coding and qualitative analysis.

All interviews were conducted and recorded with the participants’ written informed consent. The study received approval from the Research Ethics Board of the University of British Columbia in Vancouver, Canada and the Science and Ethics Committee of the Uganda Virus Research Institute and the Uganda National Council for Science and Technology in Uganda.

## Results

### Initial Demographics

All clients consented to the qualitative interview process. A total of 57 individuals (28 male and 29 female) comprising 28 partnerships were enrolled into the study. Of the 28 couples, in 22 the male member was the initially seropositive partner. The average age of all individuals involved was 42 years, with a standard deviation of 9.2 years.

### Relationships and HIV risk

During the qualitative interviews it was reported that a total of 16 (57 %) of the partnerships involved polygamous relationships; which differed from the information at recruitment where it was reported that only one couple was polygamous. The distinction between wife and extramarital relationships is sometimes difficult to discern in Ugandan communities, where co-wives often live in separate compounds and may even be sometimes unaware of each other’s existence. Indeed, some women noted that they only became aware of the presence of another wife in the relationship with the diagnosis of HIV. Most HIV-positive men cited that they stopped extramarital affairs with their HIV diagnosis. An interesting reason stated for this was the possibility of HIV “mixing”. As commented:

‘If you have sex outside marriage you can get another kind of HIV […] when they mix, the one that you have wakes up’ (Female, initially negative but seroconverted; 26 years old)

Only one woman reported having extramarital relationships. While many individuals mentioned previous relationships and marriages, men were often more open about their histories and any concurrent relationships they may have had at the time of the interview.

Another question asked of the couples regarding their sexual relationship was the adjustment of the frequency of sexual intercourse as a means of prevention. A majority of couples reduced the frequency after a positive diagnosis, and this follows some of the education they were subject to upon knowing their discordant status. Many participants reported having sex about once every week after reduction. Although abstinence is often mentioned as an option for serodiscordant couples, none of the couples said that they were abstaining. The longest duration that a couple went without sexual intercourse was approximately two months. One reason for rejecting abstinence centred on the importance of sexual intercourse as a means of maintaining the marriage bond, as cited by an HIV-positive male:

‘But the problem is that when you decide to stop having sex, then your wife will not stay home […] she would say, “My husband no longer has energy to have sex, let me leave.’ (Male, initially seropositive in lasting serodiscordance; 40 years old)

This concept of having sex to maintain the marriage for fear of losing the partner or the partner searching for other sexual partners was commonly reported among male participants. A major reason cited by female participants was that husbands would often suspect them of being unfaithful if abstinence was mentioned as an option. Only a small number of couples cited attempt to conceive as a reason for avoiding abstinence.

### Condom use and supply

Condoms were the dominant method of HIV prevention reported by the couples in this study. Other methods mentioned included female condoms and withdrawal before climax. Some couples reported some difficulties with first learning how to use condoms, but these problems were rectified soon afterwards. Preference for using condoms varied significantly by gender and by serodiscordant status. Many males expressed their distaste for using condoms, saying that they “squeeze too much”, decreased pleasure, and cause itching and pain. Some women also expressed discontent at the use of condoms, commonly citing “burning pain” in the genital areas with their use. Many seroconcordant couples had troubles using condoms consistently especially before seroconversion, mainly on part of at least one partner refusing to use them. Proper use was cited to be more difficult before seroconversion.

The hardest or most difficult thing was using condoms at the time […] we are afraid of talking about condoms and that is why I found it even hard to use them since I didn’t know how to use them properly […] some condoms were too slippery and go off […] I think it was in 2011 after I had attended the study on how to use condoms [that I started using them properly]. (Male, initially seronegative but seroconverted; 50 years old)

An interesting finding was that the couples who remained serodiscordant more commonly reported that they came to a mutual agreement on condom-use, while the couples where seroconversion occured usually left the decision to one member or chose not to use condoms at all. This indifference to condom use was often reported as being present even before seroconversion occured in these couples, although the non-adherence to condom use became more pronounced after seroconversion occurred.

Condom supply was adequate, and very few couples recalled situations when they wanted to have sex but no condoms were available. As mentioned, the main issue with condom-use was with agreement between couple members and consistent usage.

### Perceptions about discordance

When asked of their own opinions about serodiscordant status, many individuals cited confusion and disbelief. Almost all individuals said that upon initial discovery of discordance, they did not believe the test results. Participants had multiple explanations for discordance. A common explanation given was mistrust of the testing process; the phrase “testing machines were faulty” was a recurrent perception mentioned by many participants. Individuals said that the machines could have been malfunctioning, “could not be seeing the virus”, or that the testing personnel were lying to them.

Another common explanation for discordance was the possibility of resistant or strong blood types, exemplified by the frequent mention of the blood group O. One example, cited by a HIV-positive female shows the perceived differences in blood type, classified as “strong” and “light”:

‘The blood is different. You might have strong blood yet your spouse has weak blood. There is blood type A and O […] the strong one is the one of O. The one of A is lighter.’ (Female, seropositive in couple where seroconversion occurred; 30 years old)

Blood type was commonly mentioned alongside the possibility of blood being able to hide the virus from detection. The couples where seroconversion occurred appeared to report this belief more frequently, as they believed that the virus had been in their blood all along and that seroconversion was inevitable. Other explanations for discordance included “will of god” and high frequency of HIV testing, where participants saw the high frequency of testing directly contributing to the prolonged state of serodiscordance and prevention of seroconversion. Some individuals had no explanation at all for discordance, and expressed much confusion at their situation.

When asked about any advantage to being serodiscordant, a majority of couples said that the seronegative partner could continue caring for the family and children. The belief that the seropositive member would not have long to live or would not be able to lead a healthy life was widespread, despite the extensive rollout of ART in Uganda and the fact that many of the HIV positive participants had been living with the virus for over five years. This belief also contributed to some couples stringently using condoms to prevent transmission.

The few individuals who believed that discordance was possible and present were often previous participants in serodiscordant couples group offered by the TASO program in Jinja. This understanding did not come quickly or easily for these individuals, as they said that it took extensive education and counselling to convince them of the reality of discordance. Frequent testing and consistent negative results for the seronegative member also considerably helped for these individuals to believe in their discordant status.

### Community Discordance Beliefs

Individuals were also asked about their perceptions of discordance in the community. Many couples said that community members did not believe in the possibility of discordance, often dismissing it outright. The most common reaction that individuals encountered upon telling community members was that they were lying; as stated by a HIV-negative female:

‘The villagers say it is a lie […] the person is your spouse so how possible is it for one to have HIV and the other doesn’t? […] unless someone has attended the sensitizations, then they would understand that it is possible.’ (Female, seronegative in couple which remained serodiscordant; 39 years old)

Participants expressed a perceived lack of education to be a primary driving factor behind the beliefs propagated in the community. Beliefs similar to the ones held by the individuals were also mentioned, including the existence of resistant/strong blood and faulty/lying testing processes. Some seronegative individuals were advised to leave their seropositive partner by friends and villagers upon finding their discordant partners; none of them seem to have taken this advice.

Stigma regarding HIV status and discordance was still apparent in the communities; several individuals were not able to talk about communities’ perceptions of discordance as they decided not to tell anyone about their discordant status. Stigma, rumours, and gossip were cited as common reasons for choosing not to disclose. The equivalence of an HIV diagnosis to a death sentence was commonly seen from community members, as was expressed by an HIV-positive male:

‘People say that we are already dead much as we can still walk.’ (Male, seropositive in couple where seroconconversion occurred; 42 years old)

While many opinions were not as extreme, overall the attitude of villagers and communities regarding discordance were rife with distrust, and the degree of misinformation was reported to be even more severe than those reported by the study participants.

### Antiretroviral Treatment

All seropositive individuals were receiving ART prior to the interview, even though some of them had not yet initiated treatment during the course of the main HAARP study. Individuals generally showed a positive attitude to the use of ART, citing that many of them experienced a drastic increase in quality of life and health status with the start of ART treatment. Some individuals experienced side effects at the beginning of their treatment, but these eventually resolved within a few months. When asked about the impact of ART on their sex lives, most of the couples said that there were no noticeable changes in their sex lives; the remaining few said their libido decreased with the start of ART. However, they said that the reduction in frequency of intercourse may have played a role in the perceived decrease in libido.

The community’s perceptions of ART as reported by the study participants were more negative. Most individuals said that community members were distrustful of ART. Although some said that the community was thankful for the presence of ART and for the number of people who had recovered and were in good health. One individual, an HIV-positive male said:

‘When I first began taking [ART] people used to say all sorts of bad things about [it] and that it was meant for cows and other animals’ (Male, seropositive in couple where seroconversion occurred; 52 years old)

Another individual, reported that among members of her community ART indicated impending death:

‘[…] people think that when you start taking ART, you are just nearing your grave and it is given to people so that they die slowly’ (Female, initially HIV negative, but seroconverted during study; 50 years old)

This opinion was reinforced by the imagery of “spades and hoes” accompanying ART. Spades and hoes are burial tools in Uganda, and some participants noted that the ART boxes had pictures of these tools. The association of the spades and hoes and ART meant that a person taking them was close to death, and some individuals were actively dissuaded from taking the medicine.

When asked about the possibility of ART failure, opinions were divided; while some believed it could happen (and saw real instances of it happening), some had never thought about the possibility or were unable to understand the concept of treatment failure. Opinions were varied on the impact of ART on transmissibility of HIV; as expressed by the following individuals:

I personally think that [ART] increased the chances of contracting HIV. This is because it had been barely an hour after I had taken ART and what I think is that the HIV got very angry and increased its severity in attack. [My husband] therefore ended up getting it. (Female, Seropositive in couple where seroconconversion occurred; 35 years old)

[ART reduces chances of contracting HIV] because the virus becomes dormant […] If you take medicine at the prescribed time for instance at 8:00 am and again at 8:00 pm it will still be dormant and continue being dormant. (Female, Seropositive in couple which remained serodiscordant; 48 years old)

## Discussion

Through in-depth, gender-matched interviews with both members of serodiscordant couples in a rural Ugandan community, we were able to elucidate some of their perceptions about discordance in relation to their intimate relationships, ART, and prevention methods. Most participants had poor understanding of serodiscordance; most did not present scientifically sound reasons for sustained serodiscordance. Though many participants believed that this lack of scientifically sound reasoning for discordance was a driving force in misperceptions of the community, health education alone may not be sufficient to change strongly held health beliefs. Some of the reasons given, such as the existence of immune blood and the virus in hiding, have been seen in previous qualitative studies of HIV serodiscordance in Africa [18, 29, 30]. Some of these misconceptions may result in stigma and suboptimal use of prevention methods, which were cited as common reasons for seroconversion [31]. Misconceptions were common in regards to ART as well, with many individuals citing their or their community’s negative opinions of ART. Although this mistrust was cited in a study carried out in Uganda in 2004-05, [18], it is remarkable that after the intense scale-up of ART programs and HIV education over the last decade, mistrust in diagnosis and medication remains widespread and may play a role in undermining prevention measures.

Difficulties were commonly cited with the use of condoms among these couples, although most people encountered these problems only at the initiation of these methods. Disagreement in use between partners were frequent, which likely reflected complex gender roles and societal factors which determine use. Participants also reported some issues with their comfort in using condoms. Abstinence was not cited as a common prevention method used and for those who did report attempting to abstain, this did not last for long periods of time, which is consistent with findings from previous research [32]. A look into the complex factors dictating a satisfactory sexual relationship revealed that the desire for abstinence was often overshadowed by a need to minimize suspicions of unfaithfulness or a lack of virility. Although Uganda has long promoted abstinence as a part of its HIV prevention campaigns, serodiscordant couples did not seem to seriously consider it as a choice for prevention of HIV transmission.

Interestingly, some couples reported that there was a difference in stringent condom-use before and after discovering their serodiscordance. This change in sexual behavior after testing is common in African settings, especially when accompanied by counselling and education [33, 34]. We also found that the couples who seroconverted tended to report less consistent condom use before becoming seroconcordant; although this difference was not found in the quantitative analysis of the parent HAARP study [27].

Respondents reported that they believed that the community’s thoughts of HIV and treatment were more negative than those held by the couples. Participants reported that the community still perceived discordance to be impossible, and mistrust of ART was common. The community’s knowledge and perceptions play a significant role in the stigmatization of HIV and serodiscordance [35, 36]. The reported community view that an HIV infection is the equivalent of a death sentence, and that ART is given to those nearing their deaths are particularly disturbing. These perceptions likely serve as impediments to the efforts to properly educate serodiscordant couples and HIV positive individuals in sub-Saharan Africa.

There are some limitations to the study. The couples recruited into this study, as TASO clients have access to counselling regarding discordance. Participants in this sub-study have all been followed in the HAARP cohort study and were thus previously exposed to counselling about prevention, medical treatment, and serodiscordant status. Accordingly, they likely do not represent most serodiscordant heterosexual couples in rural African settings who have not participated in such programs. However, given that these individuals appear to have many misperceptions about HIV infection, it is likely that these perceptions are even more strongly held by other serodiscordant couples. Although interviewers were trained on how to elicit answers in a non-judgemental manner, some social desirability bias may exist. Participants may have been uncomfortable talking especially about extramarital relationships, which were not often disclosed by female participants during the interviews.

Much progress has been made in prevention and treatment of HIV in sub-Saharan Africa, especially in heterosexual couples where transmission is most common. However, the phenomenon of serodiscordance continues to remain poorly understood by those affected by it and the communities surrounding them in rural Ugandan settings. After extensive education campaigns and communication about HIV prevention and medication, the efforts do not seem to be penetrating deeply into rural regions, at least in terms of some of the more complex issues, such as HIV serodiscordance. Fortunately, the serodiscordant couples themselves seem to be better informed on their situations than others in the community, but these individuals still encounter scepticism when discussing their condition. A better understanding of serodiscordance is fundamental to prevention of heterosexual transmission in sub-Saharan Africa, and perceptions regarding it should be placed in the cultural context where participants live.

## Conclusions

Despite the availability of HIV treatment and education regarding serodiscordance, affected individuals (serodiscordant couples) in rural Uganda and their communities still have a poor understanding of the phenomenon and proper prevention methods. Misinformation was more prevalent in the communities more so than the couples themselves. Participants had the most difficulty explaining the reasons for serodiscordance. Most participants recognized the efficacy and importance of ARTs, but some community members remained suspicious of the drugs and treatment. Stigma about a HIV-positive status and serodiscordance remains widespread. We conclude that understanding of serodiscordance remains poor in rural areas, and this is likely to be the case in many rural communities in sub-Saharan Africa. Improved education regarding serodiscordance and ART treatment will be required to address heterosexual transmission and ensuring the maintenance of serodiscordant status in those affected.

## Abbreviations

ART: Antiretroviral treatment
HAARP: Highly Active Antiretroviral therapy as Prevention
HIV: Human immunodeficiency virus
MSM: Men who have sex with men
TASO: The AIDS Support Organisation
TasP: Treatment as prevention
VCT: Voluntary counselling and testing
